# Retrieval of Gastric Band Eroding Into the Stomach: A Gastrointestinal Fistula Case Managed Through a Combined Laparoscopic and Colonoscopic Approach

**DOI:** 10.7759/cureus.53846

**Published:** 2024-02-08

**Authors:** Ahmed Binjaloud, Ahad Alotaibi, Samar Alsubhi, Anfal Altamimi, Osamah Nafea, Zeyad Al Yousef

**Affiliations:** 1 Department of Surgery, King Saud University Medical City, King Saud University, Riyadh, SAU; 2 Department of General Surgery, King Abdulaziz Medical City, Riyadh, SAU; 3 Department of Surgery, King Fahad Medical City, Riyadh, SAU; 4 Department of Surgery, Dallah Hospital, Riyadh, SAU

**Keywords:** reservoir, endoscopy, gastrointestinal fistula, band erosion, adjustable gastric band complications

## Abstract

Obesity is an important public health concern worldwide. In Saudi Arabia, the overall prevalence of obesity has increased in both men and women in recent decades. The laparoscopic approach to bariatric surgery was first reported in the 1990s, with laparoscopic adjustable gastric banding (LAGB) developed soon after. The performance of bariatric procedures has increased rapidly in recent years, with safety and efficacy data available for the surgical treatment of obesity and related metabolic disorders. Herein, we report a challenging condition of a female patient who underwent LAGB insertion in 2013. The patient presented with a complaint of a foreign body passing through her rectum during defecation that was manually pushed back by the patient. Radiological imaging and upper/lower endoscopy confirmed the diagnosis of complete gastric band erosion into the stomach, and the reservoir with the remaining tube was observed inside the colon near the splenic flexure. This case was complicated by complete band erosion and gastrointestinal (GI) fistula formation following the delivery of her second child in January 2022. Colonic band erosion is a rare complication of LAGB. Most patients with gastric band erosion are asymptomatic or exhibit nonspecific symptoms. The definitive management of gastric band erosion involves band removal. Several approaches are commonly used in clinical practice. In our case, the band was removed using a combined laparoscopic and endoscopic retrieval approach, which is the first such report in the literature.

## Introduction

The laparoscopic approach to bariatric surgery was established in the 1990s, and in 1994, Belachew first performed laparoscopic adjustable gastric banding (LAGB) [1]. Over the last 10 years, the number of bariatric procedures has grown rapidly, representing a safe and efficient surgical treatment for obesity and related metabolic disorders [2]. LAGB is reversible and does not carry a risk of anastomotic or stapler line leakage; however, LAGB has decreased in popularity as more data on associated complications have been reported [3]. Specific complications associated with LAGB include stomach pouch enlargement, band slip, band erosion (BE), port-site infection, and port breakage [4]. BE is an uncommon complication with an incidence of approximately 1.46% [5]. A high clinical index of suspicion is necessary to diagnose BE, as most patients are asymptomatic [6]. Previous studies have reported several cases of gastric BE in the gastrointestinal (GI) tract [6-11]. Conversely, the formation of a GI fistula is an extremely rare complication that can occur simultaneously with gastric BE [12,13]. Patients in the aforementioned cases were managed with either laparotomy or laparoscopic surgery. Our report presents a rare complication of completely adjustable gastric BE in the stomach, with resulting small bowel fistula formation and reservoir penetration into the colon. We applied a combined laparoscopic and endoscopic (colonoscopic) approach to completely retrieve the bands and tubing system.

## Case presentation

**Figure 1:**
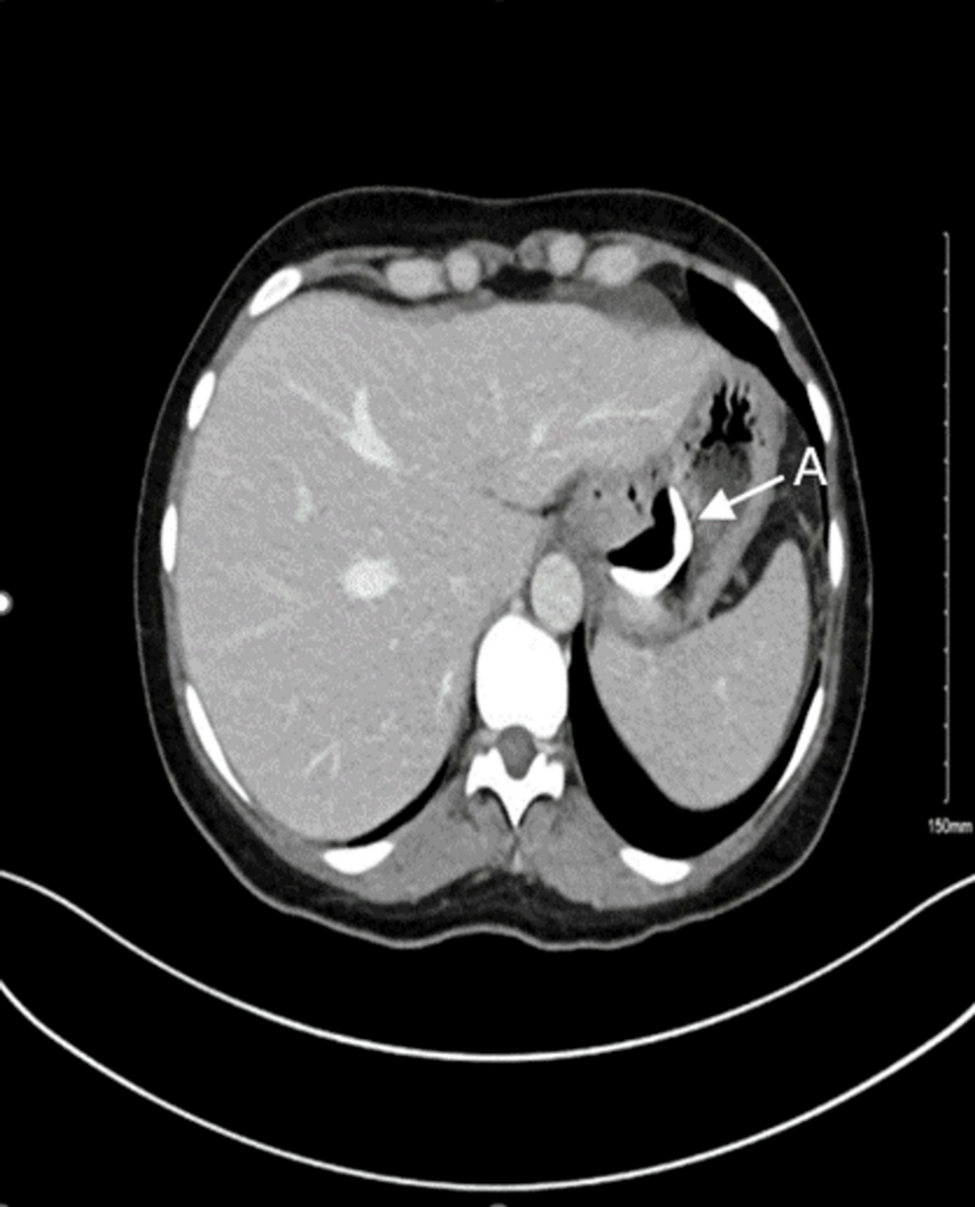
Computed tomography with contrast of the abdomen and pelvis showing findings of band eroding into the stomach (arrow A).

**Figure 2:**
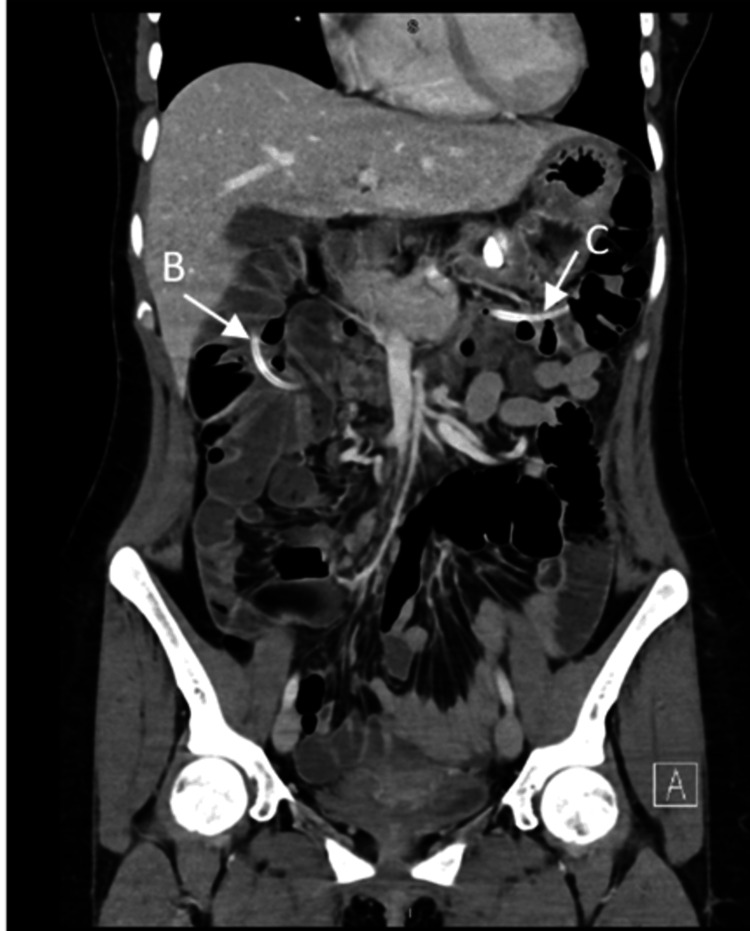
Computed tomography with contrast of the abdomen and pelvis showing findings of the tube passed into the gastrointestinal (GI) tract (arrows B and C).

A 34-year-old woman with a history of LAGB performed in 2013 presented to our clinic in January 2022, complaining of abdominal pain at the access port (reservoir). The pain had started three months after her second childbirth; at the same point, she could no longer feel the reservoir in her abdomen. She reported an episode of anal pain during defecation, followed by the observation of a hard object and a tube exiting the anus, which was pushed back in manually, and did not recur. This event occurred two weeks before her presentation. Upon examination, the patient was stable and the abdomen was soft, nondistended, and nontender. The reservoir was not palpable during the abdominal examination. Rectal examination results were unremarkable. We believe that the peristaltic movement of the bowel could have pushed the access port further down to the rectum and that the access port returned because of the stretch effect from the tubing system attached to the gastric band, which acted as a fixed point to retract it. The patient was electively admitted for further investigation. Abdominal and pelvic computed tomography (CT) revealed an intraluminal gastric band, indicating complete erosion of the tube through the anterior gastric wall into the distal ileum which may happened due to chronic pressure over the abdominal wall during pregnancy, which led to erosion of the access port, reservoir, and tubing system inside the bowel and forming a fistula between the distal part of the stomach with the ileum. The gastric band was eroded into the stomach and was observed within the lumen of the stomach embedded within the tissue as well as the presence of complete erosion of the tube through the anterior gastric wall into the distal ileum. A fistula was observed in the terminal ileum 12 cm proximal to the ileocecal valve. The tube was passed through the ileocecal valve into the cecum, ascending colon, and transverse colon, and a reservoir was observed within the splenic flexure (Figures 1-2).

**Figure 3:**
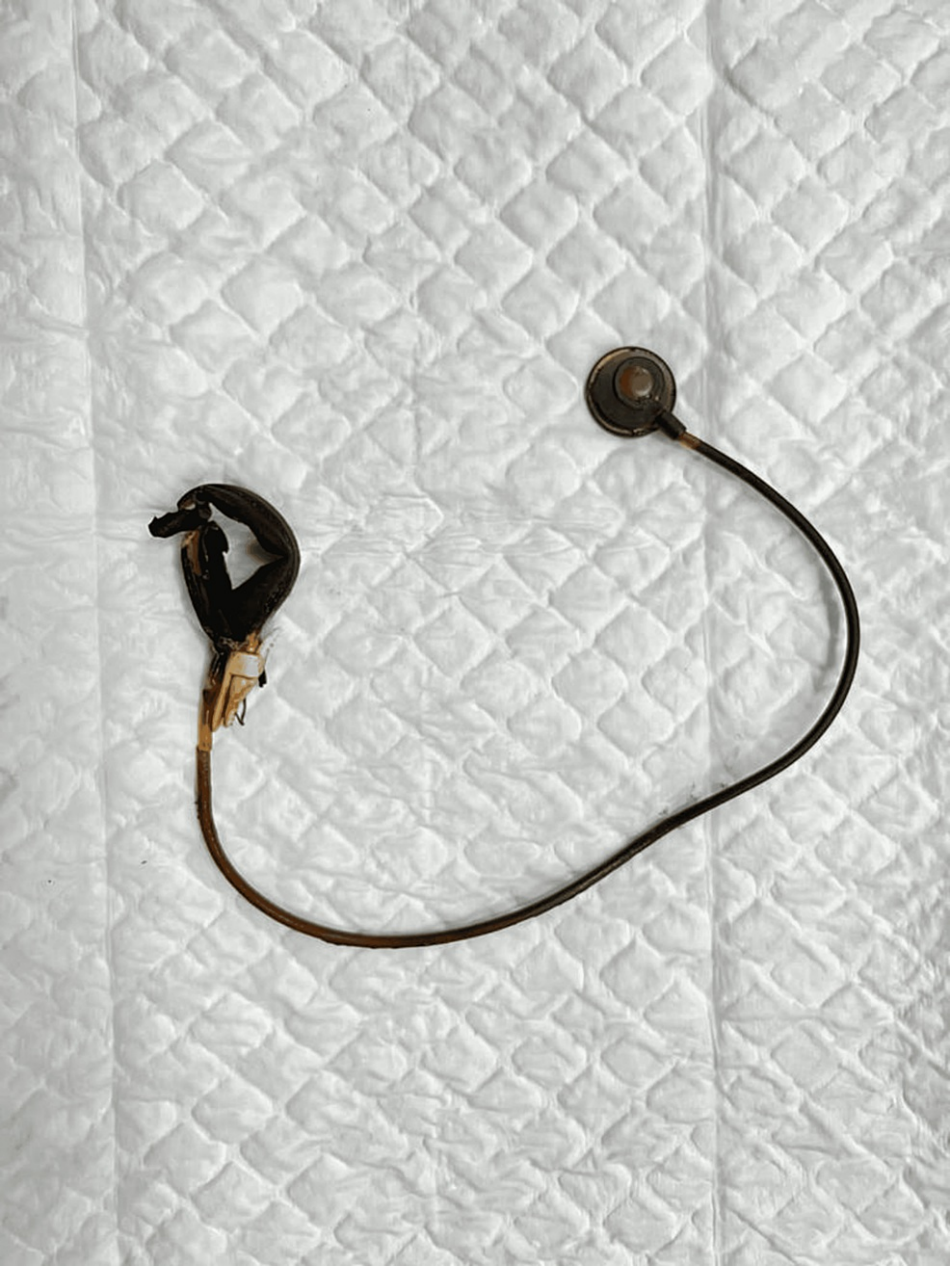
The gastric band, tubing system, and reservoir after complete retrieval. Image credit: Ahad Alotaibi

Upper endoscopy revealed gastric wall penetration at the fundus and distal stomach wall penetration through the gastric band tube. We failed to remove the band during the upper endoscopy. Preoperative colonoscopy failed to provide any relevant information, and the procedure was aborted because of poor bowel preparation. We decided to take the patient to an operating theatre to perform a diagnostic laparoscopy with intraoperative upper and lower endoscopies on the day after proper bowel preparation. The operation was initiated using upper endoscopy, and a gastric band was observed within the lumen of the stomach embedded within the tissue. Again, we attempted to remove the gastric band, but failed. Subsequently, a laparoscopic surgery was performed. Abdominal inspection revealed soft adhesions between the omentum and anterior abdominal wall, and the transverse colon was adhered to the abdominal wall in the left upper quadrant; thus, adhesiolysis was performed. Inspection around the stomach did not reveal any visible part of the band or tubing system. The cecum was observed in the right upper quadrant, with the terminal ileum near the distal stomach, and no visible fistula tract was detected. Dissection of the area was subsequently performed. However, no significant findings were observed. Thus, we elected to remove the band by opening the stomach. Therefore, a longitudinal gastrotomy was performed on the anterior wall of the stomach. Two stay statures were used to temporarily retract the stomach from the abdominal walls. The band was observed within the lumen of the stomach, and laparoscopic scissors were used to remove it through a 12-mm trocar. The tubing near the band was cut and removed from the stomach. The longitudinal gastrotomy was closed with two fires using a 60-mm Echelon blue stapler. Intraoperative colonoscopy was performed, and the reservoir with the remaining tube was observed inside the colon near the splenic flexure, which was easily grasped and removed through the rectum without resistance by the colonoscope. Subsequently, the gastric band, tubing system, and reservoir were examined to ensure complete retrieval (Figure 3).

**Figure 4:**
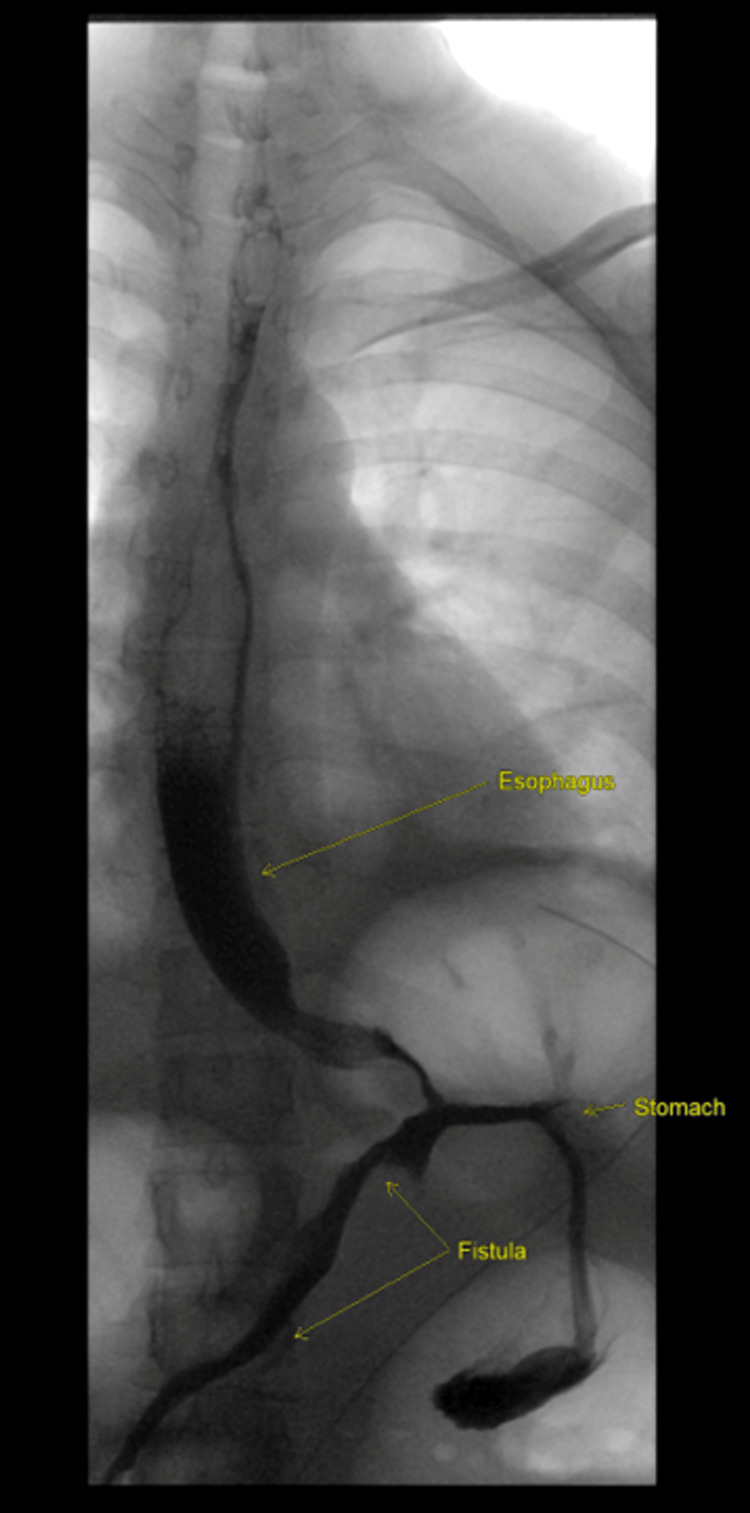
Upper gastrointestinal (GI) study indicative of a GI fistula.

After gastrostomy closure, intraoperative upper GI endoscopy was performed, which revealed no significant findings, other than that the site of the fistula and stapler over the gastrostomy showed no leak. The patient’s postoperative course was uneventful, and oral intake was gradually initiated based on the patient’s tolerance. An upper GI series was performed on the third postoperative day, and the findings were consistent with the intraoperative findings, indicating GI fistula (Figure 4). The following day, abdominal and pelvic CT with oral and rectal contrast agents did not show any intra-abdominal contrast leakage. The cecum and ileocecal junction were observed in the right upper quadrant, and a solitary GI fistulous tract was observed between the distal stomach and ileum. The next day, the patient was discharged in a stable condition and has undergone follow-up for one year postoperatively. During this period, she has remained in good condition, with no active complaints.

## Discussion

Gastric BE is a low-frequency complication of LAGB. A study conducted by Nocca et al. in 2005 revealed a gastric band migration rate of 0%-11% [14], while a later systematic review reported an incidence rate of 1.46% for gastric erosion (GE) in LAGB [5]. Colonic erosion is a rare complication associated with GB. Since the introduction of LAGB in the early 1990s, only six cases have been reported [6,12,13-16]. Gastric BE can occur within weeks or months [17]. The etiology of BE is unclear, as no scientific assessment of this complication has previously been performed [17,18]. However, Corvini et al. stated that the risk factors are multifactorial and that BE can be classified as early or late [7]. Early BE is caused by iatrogenic intraoperative gastric wall perforation, serosal injury, or the use of nonsteroidal anti-inflammatory drugs. Late BE primarily occurs at least two years after surgery and is caused by an inflammatory process provoked by the GB as a foreign body, an infected band or reservoir, or a pressure-induced response triggered by overfilling of the band, causing gastric wall ischemia. Additional factors include excessive stress on the gastric pouch owing to forced endoscopy or excessive vomiting [14]. In our case, gravid uterine pressure was thought to be the reason for the erosion of the reservoir into the colon during pregnancy. The presentation of GE is variable, ranging from completely asymptomatic to bowel obstruction and sepsis. Approximately 15%-46% of GE cases are asymptomatic [19,20]. The most common symptoms of gastric BE are abdominal pain and weight restriction [12,19,20]. Notably, none of the previously reported cases involved the passage of the tube system through the rectum before hospital presentation, as in our patient, except for one reported case in 2012 of unusual transient small bowel obstruction, followed by complete passage of the gastric band and its connecting system through the rectum without further intervention [21]. Usually, when BE is suspected, plain radiography is performed to confirm the diagnosis. Moreover, endoscopy is an important tool in the diagnosis and treatment of BE, as it helps in diagnosing the stage of erosion, which can be classified into three stages: stage I, the GB can be recognized through a hole in the gastric wall; stage II, partial migration of the GB into the gastric lumen; and stage III, complete migration of the band and tube into the gastric lumen [14]. In a prospective study conducted by Silecchia et al., 123 patients were followed up for between 12 months and four years [22]. Routine endoscopy has been shown to detect 7.5% of asymptomatic GE cases [18], indicating that the incidence of GE could be underestimated and that a longer follow-up period may reveal a higher rate of GE [10]. Band removal is the most common management strategy for GE and can be performed either laparoscopically or endoscopically, both of which are safe and feasible [5,22,23]. For the endoscopic approach, Neto et al. advised waiting until the erosion reached >50% of the band circumference for easier removal [20]. However, this approach may require multiple endoscopies, resulting in delays in GB removal [5]. In addition, the endoscopic approach has a long procedural time and a high failure rate if the band is embedded in the gastric wall [5]. Therefore, current recommendations support performing the laparoscopic approach over endoscopic removal [5]. In this study, we used the laparoscopic approach, as it provides the advantages of adhesiolysis, bowel exploration, band removal, immediate repair, and possible repair of the fistula, which are not possible with the endoscopic approach. In this case, the band was removed laparoscopically as it was embedded within the stomach tissue, while the reservoir and tube systems were removed via colonoscopy as they were intraluminal (distal ileum and colon). Regarding the GI fistula between the stomach and distal ileum, which was reported preoperatively on CT and observed intraoperatively, the decision in the operation theatre was not to proceed with further dissection and to remove the fistula due to the massive adhesions, as well as the distorted anatomy, we encountered. Subsequently, we found that the terminal ileum and cecum were pulled up toward the right upper quadrant near the fistula site by the tubing system. During the surgery, we agreed to remove the foreign body (i.e., the tubing system) from the fistulous tract by removing the whole gastric band parts to facilitate the closure of the fistula tract, as the presence of a foreign body within the fistula tract would not help in closing it. We weighed the risk of injury to vital structures such as the duodenum or biliary ducts during dissection against the risk of malabsorption from the fistula tract. Therefore, we aimed to achieve spontaneous closure of the tract after removing the foreign body, as the other risk factors for unhealing the fistula, such as steroid usage or radiation, were not present in this patient.

## Conclusions

In our case, on top of the eroded gastric band, the access port was not in the site where it was placed in the primary surgery, which is the anterior abdominal fascia; it was found inside the colon. We assumed after two pregnancies and the chronic pressure over the abdominal wall, this has led to erosion of the access port inside the colon. The formation of adhesions between the colon and the abdominal wall is in favor of supporting our assumption. The first step was to try to remove it by the endoscopic approach. We did upper and lower GI endoscopies under mild sedation to assess and try to retrieve the gastric band and the access port. Unfortunately, we failed due to the chronic fibrosis around the band and the tube, which was going out of the distal stomach, and the presence of the fistula in upper GI endoscopy as well as the radiological exams. Removal of the access port by endoscopic approach alone has failed too. The decision was to not proceed with more trials of the endoscopic approach and no need for further images as the diagnosis was made clearly by the CT scan and the upper GI scope. The decision was made to do a dual approach in the surgical theater under general anesthesia by laparoscopic and endoscopic techniques to make sure that the gastric band is removed entirely from the GI tract, assess the fistula site, and get a better view of the whole abdominal cavity as well as recheck the stomach at the end of procedure making sure no leak from the gastrotomy site closure. The surgical team agreed to not proceed with further dissection or attempt to remove the fistula due to the massive adhesions, as well as the distorted anatomy. We agreed to remove the foreign body from the fistulous tract by removing the whole gastric band parts to facilitate the closure of the fistula tract, as the presence of a foreign body within the fistula tract would not help in closing it. We weighed the risk of injury to vital structures such as the duodenum or biliary ducts during dissection against the risk of malabsorption from the fistula tract. Therefore, we aimed to achieve spontaneous closure of the tract.
